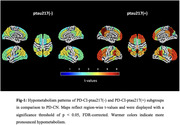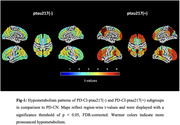# The effect of AD co‐pathology on cognitive phenotype and FDG‐PET patterns in Parkinson’s disease with cognitive impairment

**DOI:** 10.1002/alz.087594

**Published:** 2025-01-09

**Authors:** Sandra Castro‐Labrador, Jesús Silva‐Rodríguez, Miguel Labrador‐Espinosa, Laura Muñoz‐Delgado, Pablo Franco‐Rosado, Ana María Castellano Guerrero, Daniel Macías‐García, Silvia Jesús Maestre, Astrid Adarmes‐Gómez, Elena Ojeda‐Lepe, Fátima Carillo, Juan Francisco Martín Rodríguez, San Eufrasio M, Cristina Pérez‐Calvo, Nicholas J. Ashton, Henrik Zetterberg, Florinda Roldan, David Garcia‐Solis, Pablo Mir, Michel J. Grothe

**Affiliations:** ^1^ Reina Sofia Alzheimer Center, CIEN Foundation, ISCIII, Madrid Spain; ^2^ Centro de Investigación Biomédica en Red sobre Enfermedades Neurodegenerativas, Instituto de Salud Carlos III, Madrid Spain; ^3^ Wallenberg Centre for Molecular and Translational Medicine, University of Gothenburg, Gothenburg Sweden; ^4^ Unidad de Trastornos del Movimiento, Servicio de Neurología y Neurofisiología Clínica, Instituto de Biomedicina de Sevilla, Hospital Universitario Virgen del Rocío/CSIC/Universidad de Sevilla, Seville Spain; ^5^ Clinical Neurochemistry Laboratory, Sahlgrenska University Hospital, Mölndal Sweden; ^6^ Department of Psychiatry and Neurochemistry, Institute of Neuroscience and Physiology, The Sahlgrenska Academy, University of Gothenburg, Mölndal, Gothenburg Sweden; ^7^ Department of Psychiatry and Neurochemistry, Institute of Neuroscience and Physiology, the Sahlgrenska Academy at the University of Gothenburg, Mölndal Sweden; ^8^ Department of Psychiatry and Neurochemistry, Institute of Neuroscience and Physiology, The Sahlgrenska Academy at the University of Gothenburg, Mölndal Sweden; ^9^ Unidad de Radiodiagnóstico, Hospital Universitario Virgen del Rocío, Seville Spain; ^10^ Unidad de Medicina Nuclear, Hospital Universitario Virgen del Rocío, Seville Spain; ^11^ Departamento de Medicina, Facultad de Medicina, Universidad de Sevilla, Seville Spain; ^12^ Reina Sofia Alzheimer Centre, CIEN Foundation, ISCIII, Madrid Spain

## Abstract

**Background:**

Co‐morbid Alzheimer’s disease (AD) pathology is a major risk factor for cognitive impairment (CI) in PD, but whether and how AD co‐pathology affects the clinical phenotype of PD‐CI is incompletely understood. Recently validated plasma biomarkers for AD pathology, such as ptau217, hold great promise to revolutionize the diagnosis of neurodegenerative diseases. Here, we used plasma ptau217 to detect AD co‐pathology in a well‐characterized cohort of PD patients with CI and examine its associations with APOE4 genotype, cognitive profile, and cerebral hypometabolism on FDG‐PET.

**Method:**

Eighty‐eight PD patients were stratified into PD‐CI (N=50; 24 PD‐MCI, 26 PDD) and PD with normal cognition (PD‐CN; N=38) using neuropsychological testing with the PD‐Cognitive Rating Scale. All patients had a blood draw and an FDG‐PET scan at study inclusion. Plasma ptau217 levels were measured using the ALZpath ptau217 Simoa immunoassay, and patients were classified as ptau217(+) and ptau217(‐) using an established threshold (0.4 pg/mL). APOE4 alleles were genotyped and coded as a binary variable. FDG‐PET data was processed using SPM12 and brain‐wide hypometabolism patterns (vs PD‐CN) were assessed across 52 atlas‐defined brain regions. In addition, we explicitly tested whether PD‐CI‐ptau217(+) had specifically more pronounced hypometabolism in an a‐priori region‐of‐interest (ROI) composed of temporo‐parietal areas typically affected in AD.

**Result:**

Fourteen PD‐CI (28%) and 5 PD‐CN (13%) were classified as ptau217(+). PD‐CN‐ptau217(+) were excluded from further analyses. Compared to PD‐CI‐ptau217(‐), PD‐CI‐ptau217(+) had a higher prevalence of APOE4 carriers (50% vs 16%, p=0.04) and more impaired memory scores (p=0.03), although global cognition (MoCA) was not significantly different (p=0.10) (Table 1). When compared to PD‐CN, both PD‐CI‐ptau217(‐) and PD‐CI‐ptau217(+) showed significant hypometabolism in posterior‐occipital, temporal, and frontal areas (p<0.05, FDR‐corrected), but hypometabolism in PD‐CI‐ptau217(+) was considerably more extensive, particularly in temporo‐parietal areas (Fig‐1). ROI‐based analysis confirmed significantly more pronounced hypometabolism of AD‐related regions in PD‐CI‐ptau217(+) compared to PD‐CI‐ptau217(‐) (p=0.01), whereas occipital hypometabolism, typical for PD‐CI, did not differ (p=0.83).

**Conclusion:**

AD co‐pathology results in a more memory‐predominant cognitive profile and AD‐like neurodegeneration phenotype in PD‐CI. Novel plasma biomarkers may significantly facilitate clinical detection of AD co‐pathology, which may have important implications for personalized diagnosis, prognosis, and treatment of PD patients.